# Recurrent Neural Network and Reinforcement Learning Model for COVID-19 Prediction

**DOI:** 10.3389/fpubh.2021.744100

**Published:** 2021-10-04

**Authors:** R. Lakshmana Kumar, Firoz Khan, Sadia Din, Shahab S. Band, Amir Mosavi, Ebuka Ibeke

**Affiliations:** ^1^Department of Computer Applications, Hindusthan College of Engineering and Technology, Coimbatore, India; ^2^Dubai Men's College, Higher Colleges of Technology, Dubai, United Arab Emirates; ^3^Department of Information and Communication Engineering, Yeung University, Gyeongsan, South Korea; ^4^Future Technology Research Center, College of Future, National Yunlin University of Science and Technology, Douliu, Taiwan; ^5^Faculty of Civil Engineering, Technische Universität Dresden, Dresden, Germany; ^6^John von Neumann Faculty of Informatics, Obuda University, Budapest, Hungary; ^7^School of Creative and Cultural Business, Robert Gordon University, Aberdeen, United Kingdom

**Keywords:** COVID-19, deep learning, LSTM, RNN, prediction reinforcement learning

## Abstract

Detection and prediction of the novel Coronavirus present new challenges for the medical research community due to its widespread across the globe. Methods driven by Artificial Intelligence can help predict specific parameters, hazards, and outcomes of such a pandemic. Recently, deep learning-based approaches have proven a novel opportunity to determine various difficulties in prediction. In this work, two learning algorithms, namely deep learning and reinforcement learning, were developed to forecast COVID-19. This article constructs a model using Recurrent Neural Networks (RNN), particularly the Modified Long Short-Term Memory (MLSTM) model, to forecast the count of newly affected individuals, losses, and cures in the following few days. This study also suggests deep learning reinforcement to optimize COVID-19's predictive outcome based on symptoms. Real-world data was utilized to analyze the success of the suggested system. The findings show that the established approach promises prognosticating outcomes concerning the current COVID-19 pandemic and outperformed the Long Short-Term Memory (LSTM) model and the Machine Learning model, Logistic Regresion (LR) in terms of error rate.

## Introduction

With the spread of the unfamiliar Coronavirus (COVID-19), which was first discovered in Wuhan city in China in 2019, societies worldwide continue to face very distressing times. On March 11, 2020, the World Health Organization (WHO) flagged the COVID-19 as a pandemic, exceeding 118,000 cases in over 110 countries. The epidemic has quickly spread through many countries, including Italy, Spain, France, the United States, and India, wreaking havoc on healthcare systems (1). Modeling and predicting the expanse of verified and recovered COVID-19 cases accurately is critical for understanding and helping decision-makers to slow down or arrest its progression. Since the COVID-19 pandemic has shifted into a global pandemic, there is a necessity for real-time epidemiological data examinations to provide the population with a strong course of action to combat the infection. Following the novel COVID-19, the world has been restlessly battling its cause (2). As of August 27, 2020, there were 24,631,906 confirmed cases worldwide, of which 17,089,939 recovered, and 841,310 ended in death (3). Table 1 shows the topmost countries affected. The COVID-19 relate itself to the species as that of SARS-CoV and MERS-CoV.

**Table 1:** Top 5 affected countries COVID-19 data.

**Country**	**Cases**	**Deaths**	**Recovered**	**Fatality (%)**
USA	28,765,423	511,133	18,973,190	1.8
India	11,005,850	156,418	10,699,410	1.4
Brazil	10,168,174	246,560	9,095,483	2.4
Russia	4,164,726	83,293	3,713,445	2.0
UK	4,115,509	120,580	2,494,218	2.96

Table 2 shows the comparison, where the symptoms initially appear as a common cold then progress to those of respiratory diseases that cause breathing problems, tiredness, fever, and dry cough. Once a large-scale break out of a contagious disease occurs and a significant public health emergency ensues, researchers use outbreak models to evaluate and forecast the disease's development pattern and determine direct measures to prevent and restraint based on the effects of the analysis.

**Table 2:** Top 5 affected countries COVID-19 data.

**Name**	**COVID-19**	**MERS**	**SARS**
Year	2019	2012	2003
Confirmed	110,927,514	2,494	8,096
Deaths	2,454,587	858	774
Fatality %	2.21	34.1	9.6
Countries	220	27	26

The most frequently used conventional pandemic schemes are susceptible—infected—recovered (SIR), and susceptible—exposed—infected—recovered (SEIR) models (4), where “S,” “E,” “I,” and “R” signify every number of susceptive persons, the magnitude of individuals during the incubation phase, the magnitude of contagious persons and the number of individuals improved, respectively. These models are trained to forecast multiple diseases, such as Ebola and SARS, due to their robust predictive abilities of the linked indications. With the emergence and spread of COVID-19, the significant research challenge is arresting the growth patterns of the spread of this disease which has been observed in several science fields throughout the globe. Thereby, different approaches (5) to modeling, estimating, and forecasting are implemented to understand and control this pandemic. Traditional disease models measure the rate of infection based on the complex variation in the number of contaminations and then determine the disease's spread and evolution pattern. Yet, those approaches assume that all individuals with Coronavirus hold an equal chance of infection, and hence, their predictive results can only suggest general patterns and are restricted.

Artificial Intelligence (AI) is lately being applied toward stimulating biomedical study and toward numerous fields such as image identification, object categorization, image segmentation, and deep learning approaches (6). For example, individuals affected with COVID-19 will possibly have pneumonia since the infection reaches the lungs. Many deep learning investigations identify the condition using X-ray images of the chest (7). Three different deep learning models (8) have been employed in the past to distribute X-ray images of pneumonia, and those are the fine-tuned model, the non-fine-tuned and the scratch-trained model. On the other hand, most prediction models use X-ray and CT images (9) based on the deep learning method, which requires more time to extract the features and train the model.

Famous classical mathematical differential equations and population prediction models have limitations on predicting the population in the time-series and significant estimation errors. Analytical methodologies, for instance, Auto Regressive Moving Average (ARIMA), Moving average (MA), and Auto-Regressive (AR) methods, are primarily formulated on the premises. Still, they have difficulties in predicting live circulation rates. A vast variety of demographic and computative models (10) were developed for modeling COVID-19's rampant transmission dynamics. However, in multiple situations, these approaches don't adhere to the provided information, and the accuracy of the forecast is usually low. Therefore, this work investigates the modified LSTM approach to forecasting the likely COVID-19 cases and deaths. It also describes deep reinforcement learning for optimizing the prediction results based on symptoms. Experiments using real data and various metrics reveal the improved performance of the work. The specific contributions of this paper include:

- Deep and reinforcement learning to predict COVID-19,
- LSTM model modified with new activation function for efficient prediction,
- Deep reinforcement learning applied to optimize results based on COVID-19 symptoms

The remainder of the paper is ordered as follows. Section related work reviews the related work. Preliminary information regarding the used approaches and the problem statement is given in section methodological preliminaries, while section optimized prediction of covid-19 describes the proposed method. The experiential details, evaluation criteria, and performance comparison are given in section optimized prediction of covid-19, along with an analysis of the conclusions attained. Lastly, section results and discussions gives some concluding observations.

## Related Work

### COVID-19 Prediction and Forecasting

Various prediction techniques that are regularly used to tackle forecasting problems include Machine Learning (ML) models (4), which can be employed toward determining the number of potential COVID-19 infected patients. Rustam et al. (4) used four simple statistical models: Linear Regression, Least Absolute Shrinkage and Selection Operator, Support Vector Machine, and Exponential Smoothing to forecast threatening COVID-19 factors. Petropoulos and Makridakis (11) presented an analytical procedure to forecast the continuation of COVID-19. The work presents a timeline of a live forecasting activity with significant possible planning and decision-making consequences and offers realistic forecasts for confirmed COVID-19 cases. Malavika et al. (12) adopted a logistic growth curve model for short-term prediction of COVID-19, and SIR models was employed in identifying the highest possible live individuals and peak seasons. In addition, the Time Disrupted Regression model is used to estimate the influence of lockdown and other important proposals.

Pal et al. (13), combined medical data with the trend and local weather data to forecast each country's level of risk. Specifically, a shallow LSTM neural network is employed in solving difficulties in limited datasets, and a country's risk level (high, medium, and recovery) is categorized using the Fuzzy rule. Hu et al. (14) used Coronavirus-specific dataset to fine-tune the pre-trained multi-task deep model. The re-trained prototype was then utilized to decide possible commercial medications upon targeted proteins of SARS-CoV-2. Finally, Salgotra et al. (9) developed Genetic programming (GP) prediction models for confirmed individuals and death cases across three of the most affected states, namely Maharashtra, Gujarat, Delhi, and India. The predictive models are expressed utilizing the specific formula, and predictive powerless variables were studied.

Velásquez and Lara (1) analyzed historical and expected COVID-19 death infections based upon the Reduced-Space Gaussian Process Regression correlated with disordered Dynamical Systems. COVID-19 forecasted with Gaussian models mean-field models can be meaningfully applied to obtain a quantitative summary of virus spread with contamination, death, and recovery rates. Jia et al. (10) adopted Logistic, Bertalanffy, and Gompertz models to prove the validity of the current statistical models by fitting and analyzing the SARS epidemic patterns. The findings were then used to fit and evaluate the COVID-19 scenarios. The forecasted outcomes of the three different mathematical models varied for different parameters and in different regions. Kavadi et al., (15) proposed partial derivative regression and a non-linear machine learning system toward the global pandemic verification of COVID-19. Dehesh et al., (16) considered the best predictive models for regularly reported individuals in nations with a huge magnitude of verified cases and then made predictions based on those models to better prepare healthcare systems. For predicting the pattern of reported events, the Auto-Regressive Integrated Moving Average model was used. Ngabo et al. (17) proposed an artificial intelligence (AI) algorithm that predicts the survival rate of COVID-19 patients based on their immune system, exercise rate, and age quantiles.

### Deep Learning for COVID-19

Arora et al. (18) used deep learning-based models to forecast the number of recorded positive cases of novel Coronavirus (COVID-19) for 32 Indian states and union territories. Recurrent Neural Network (RNN)-based LSTM variants, such as Deep LSTM, Convolutional LSTM and Bi-directional LSTM, were applied to the Indian dataset to forecast the number of positive cases. Huang et al. (19) suggest that Convolutional Neural Network (CNN) can accurately estimate and determine the number of verified cases. The emphasis was on various towns with the most reported cases in China, and a COVID-19 prognostication model was suggested based upon the CNN system of Deep Neural Network (DNN). Three deep learning models (20), namely DNN, LSTM, and CNN, were stacked in learning models for the ensemble to achieve the most reliable results. The meta-learners used these forecasted values of these models as inputs to produce the final prediction of outbreaks. Ramchandani et al. (21) proposed a deep learning model to forecast the range of increase in COVID-19 and offer an unusual approach for determining equidimensional multivariate time scale illustrations and multivariate spatial time scale results.

Yoo et al. (22) examined the usefulness of applying a deep learning-based decision-tree classifier to distinguish COVID-19 from CXR images. This classifier consists of three binary decision trees, each trained by a deep learning model based on the PyTorch system with a neural convolution network. The primary decision tree divides the CXR images as either regular or anomalous. The second tree recognizes the irregular images bearing symptoms of tuberculosis. The final tree identifies the signs of COVID-19. Ozturk et al. (23) introduced a different approach for automated COVID-19 detection employing raw X-ray images of the chest. The developed system offers honest diagnostics for binary (COVID-19 vs. No-Findings) and multi-class (COVID-19 vs. Pneumonia vs. No-Findings) classification. Panwar et al., (24) proposed a deep learning neural network-based approach nCOVnet, which employs an alternative rapid screening system to identify COVID-19 by analyzing patients' X- rays to check for visual markers present in COVID-19 patients' chest radiography images.

Hu et al. (25) proposed the weakly controlled deep learning strategy for recognizing and distinguishing COVID-19 contagion from computed tomography (CT) images. This approach reduces the manual labeling requirements for CT images, reliably diagnose infection, and distinguish COVID- 19 from non-COVID-19 cases. Deep learning-based research about CT in the chest has proven to be reliable and effective for determining COVID-19. Mohammed et al. (26) proposed ResNext+, which offers an end-to-end semi-supervised strategy to COVID-19 discovery, including data labels at volume level only, and can provide a slice level prediction. A deep, long-term bidirectional memory network with a mixed density network (DBM) model was established by Pathak et al. (27), namely the Memetic Adaptive Differential Evolution (MADE) algorithm which can fine-tune the hyperparameters for the DBM model.

### Deep Reinforcement Learning

Reinforcement Learning (RL) is a machine-learning model, where agents learn efficient techniques from trial-and-error encounters with their surroundings that produce the single most massive, long-term reward. The Q-learning algorithm (28) is the most descriptive of the RL algorithms. Q-learning can learn an acceptable method without an environmental operating prototype by modifying an action-value algorithm called the Q function. When the state-action space is large and complex, deep neural networks can approximate the Q-equation, and the corresponding algorithm is called Deep Reinforcement Learning (DRL) (29). This has promising application for rational decision-making in diverse fields, such as energy management, robotics, agriculture, healthcare, etc. This model successfully resolved a wide range of complicated decision-making assignments which were earlier outside the machine's limits.

Wang et al. (30) offered an adaptive design that relies on graph embedding in the training process during state representation and reinforcement learning. Depending on a couple of real-life datasets, the findings show that the scheme can beneficially decrease the infection's epidemiological replication rate. This approach can aid in the initial exposure of COVID-19, whereby RL may represent an effective method to combat the spread of an outbreak. Dell'Aversana (23) combines multi-layer Artificial Neural Networks with Reinforcement Learning architecture to allow software-defined factors to acquire environmentally optimized functioning. Iwendi et al., (31) utilized COVID-19 patients' geographical and demographic data to predict the severity of cases, recovery, and death. In (32) a semantic privacy framework that uses sensitive and semantically related terms to sanitize healthcare documents was proposed, and (33) uses deep learning to detect and sanitize social media comments. In (34), the authors discussed the concepts of an incentive approach for COVID-19 planning using Blockchain Technology. Deep learning and medical image processing for Coronavirus (COVID-19) pandemic were analyzed by the authors in (35) and the results are nicely presented.

## Methodological Preliminaries

This section describes the COVID-19 prediction problem and gives information about the technical background of DL and RL used within this work.

### Problem Formulation

Time scale forecasting aims to utilize the input sequences witnessed earlier to forecast a fixed-length series of expected time scale values. In machine learning, a part of the input time-series sequence, i.e., delayed values, is replaced to assist the input functions. The number of leading time levels is recognized as the width/size of the frame. Provided with a single variable time-series:


(1) $$\begin{array}{l} TS\left(t\right)=\left\{s_{1},s_{2},s_{3},\ldots ,s_{t}\right\} \end{array}$$

the intention is to forecast the future k values of the sequence, ŷ = ŷ_1_, ŷ_2_, ŷ_3_, …, ŷ_*k*_ ≅ (*s*_*t*+1_, *s*_*t*+2_, *s*_*t*+3_, …, *s*_*t*+*k*_) utilizing the values of former conclusions.

### Long Short-Term Memory Network

RNN is one of the deep learning techniques which automatically selects appropriate characteristics from the practice specimens and later supplies activation from the previous time step as information for the current time step and network's self-connections. RNN is proper for data processing and has outstanding potential in time-series forecast by saving extensive historical data in its inner state. Still, it has the limitation of disappearing and gradient-exploding difficulties, which leads to an extended practice period or practice that does not work. In 1997, Hochreiter and Schmidhuber (36) devised a long short-term memory structure to determine long-term dependence on the multiplicative passages that coordinate information and memory cells movement in the recurrent hidden layer.

**Figure 1:**
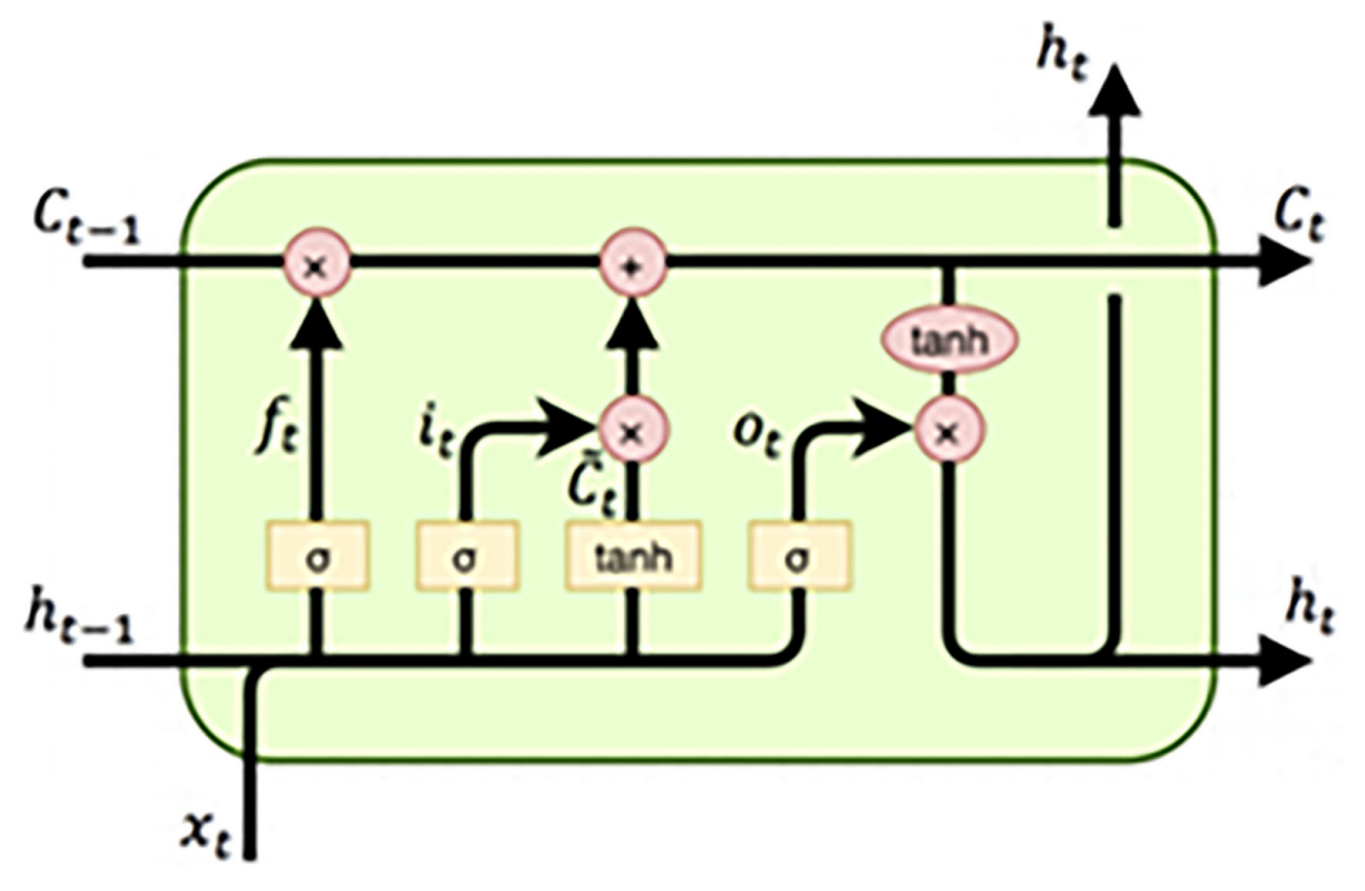
The structure of the LSTM.

Figure 1 exhibits an LSTM memory cell's primary arrangement with two distinct components: (C_t_) and the short-term state component (h_t_).

The structure of LSTM consists of the following gates: input, forget, control, and output gate. The input gate determines which information can be passed on to the cell and is defined as:


(2) $$\begin{array}{l} i_{t}=\sigma W_{i}\times h_{t-1}x_{t}+b_{i} \end{array}$$

The information to be ignored from the previous memory input is determined by the forget gate and is defined as:


(3) $$\begin{array}{l} f_{t}=\sigma W_{f}\times h_{t-1}x_{t}+b_{f} \end{array}$$

The cell update is controlled by the control gate, based on the following equations:


(4) $$\begin{array}{l} \tilde{C_{t}}=\tanh W_{c}\tilde{\times }h_{t-1}X_{t}+b_{f} \end{array}$$

(5) $$\begin{array}{l} C_{t}=F_{t}^{*}{\tilde{C}}_{t-1}+i_{t}^{*}\tilde{C_{t}} \end{array}$$

The hidden layer (*h*_*t*−1_) is updated by the output layer, which is also responsible for updating the output gate as is given by:


(6) $$\begin{array}{l} O_{t}=\sigma W_{o}\times h_{t-1}x_{t}+b_{o} \end{array}$$

In the above equation, *W* and *b* represent the weight matrix and bias vector, respectively; tanh is used to scale the values in the range −1 to 1, and σ denotes a standard logistic sigmoid function. The variables *i, f, o*, and *c* are the input gate, forget gate, output gate, and cell activation vector.

### Reinforcement Learning

**Figure 2:**
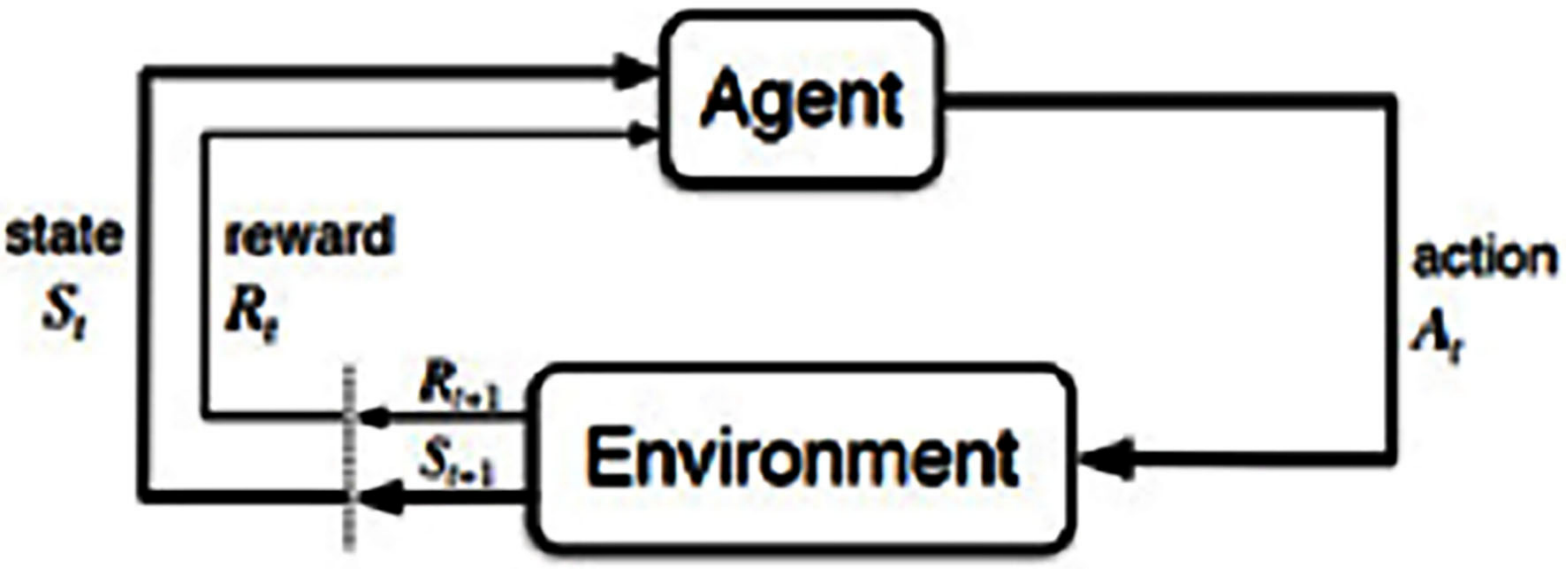
Interaction process of reinforcement learning.

Reinforcement learning (RL) is an artificial intelligence model with a progressive programming guide instructing algorithms to apply an award and penalty strategy. A Markov choice system is designated for the RL study process, which endorses the formalism of reinforcement learning difficulties. The RL algorithm is an agent that receives through communicating and associating with society. The agent will obtain incentives for the appropriate steps and penalties for the inaccurate performances. Without individual intervention, the agent learns by itself via improving his incentives and reducing his punishments. The intercommunication process between the agent and environment of RL is shown in Figure 2.

An agent available in a state (S) executes an action (A). The agent collects a reward R(S, A) for acting and shifts into a different state. A policy (π) means any mapping function of the states and actions that decide an agent's action in every state. The central goal in an agent's existence is to obtain an optimal strategy π^*^ for which the overall reduced incentive is expanded. The optimal system π^*^ is defined in equation (7):


(7) $$\begin{array}{l} \pi^{*}\left(S\right)=argmax_{a\epsilon A}\gamma \underset{s^{\prime }\epsilon S}{\sum }P_{sa}\left(s^{\prime },a\right)V^{*}\left(s^{\prime },a\right) \end{array}$$

The best assessment function is obtained from the most dependable optimal plan; it is defined through some benefits an agent receives from all the other states. The aforementioned function with optimum power is expressed in equation (8):


(8) $$\begin{array}{l} V^{*}\left(s,a\right)=R\left(s,a\right)+max_{a\epsilon A}\gamma \underset{s^{\prime }\epsilon S}{\sum }P_{sa}\left(s^{\prime },a\right)V^{*}\left(s^{\prime },a\right) \end{array}$$

Thus, a reinforcement learning agent learns through experiences with the environment. Using complex programming functions, agents optimize their compensations through estimating individual fittest optimal strategy and power function for the bellman.

## Optimized Prediction of COVID-19

The presented research investigates predictions for the novel COVID-19 using the discussed reinforcement learning. COVID-19 has proved to be a serious threat to people of all ages all over the world. It has resulted in tens of thousands of deaths so far, with the mortality rate continuing to rise regularly. This research sought to assess future estimates of the death rate, the number of infected individuals reported every day and the number of healing cases in the following 15 days to add to the pandemic's tracking. The forecasting was done using a Deep Learning framework that was designed specifically for this study.

AI is a growing platform with many different intelligent applications, such as Machine Learning (ML) and Deep Learning (DL). ML indicates the capability to acquire and deduce significant patterns of these data; furthermore, ML-based algorithms and practices' accomplishment depends heavily on the individual functions. Meanwhile, by learning from simple representation, DL can solve complex systems. DL possesses a pair of critical features (14): (1) the capacity to acquire the correct phrases and (2) the ability to help the machine discover data by sequentially using several layers to understand more meaningful representations.

**Figure 3:**
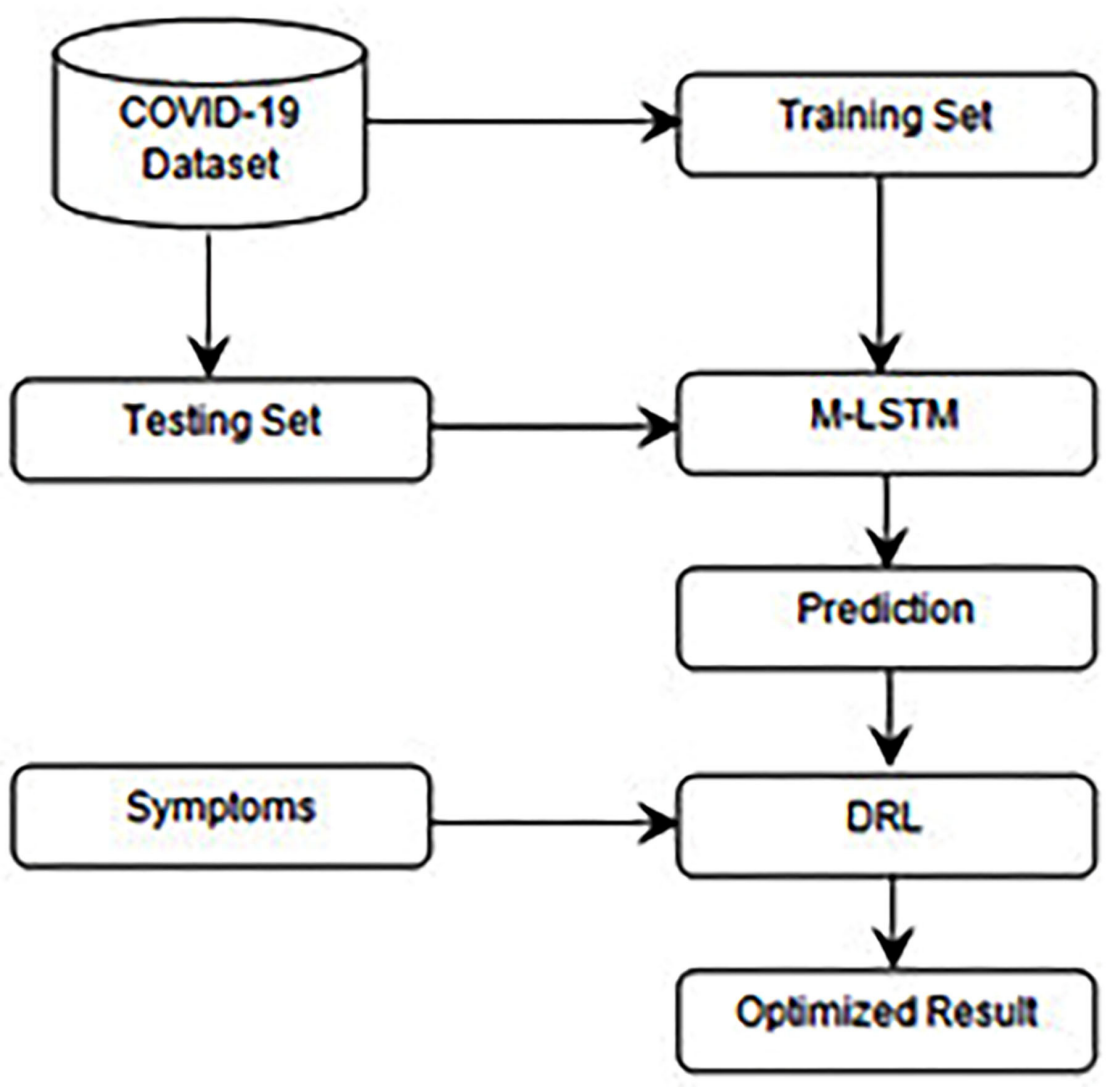
Workflow of the proposed approach.

This paper proposes an optimized prognostication of COVID-19 employing deep reinforcement learning (DRL). Primarily, Modified-LSTM was utilized to forecast the figures of verified cases and death cases. Next, the forecasted outcomes were optimized by employing DRL based on the symptoms. Figure 3 shows the proposed workflow.

LSTM is an RNN deformation structure, where the memory cell is added to the hidden layer to restrain the memory data of the timeline data. Data is transmitted through several controllable gates between different cells of the hidden layer, thus allowing control of the memory and ignoring the amount of the former and prevailing data. Two LSTM gates are designed to control the status of the memory cell. One is the forget gate, which shows how much “memory” can be preserved from the last moment of the cell; the second is the input gate, which specifies how much information about the current time can be kept to the cell status and regulates the fusion ratio of “old” data and “present” incentive. Lastly, LSTM's output gate is designed to control the extent of cell status information outputs. The structure of LSTM can be found in Figure 1.

Herein, two activation functions of LSTM are used: linear and non-linear. The traditional LSTM uses the non-linear tanh function. For best results, select the best activation function.


(9) $$\begin{array}{l} f\left(x_{i}\right)=\frac{\exp\left(x_{i}\right)}{\sum_{j}\exp\left(x_{j}\right)} \end{array}$$

Deep learning stimulates the improvement in RL, whereby DL algorithms within RL describe the deep reinforcement learning (DRL) area. Deep learning allows RL to scale up the earlier unmanageable issues, i.e., settings by a high-dimensional state, areas for the interruption, and decision-making. Deep reinforcement training employs a deep neural network to approximate every reinforcement learning function, including value function, Q function, transformation system, and reward function. Q-Learning is an RL system that decides which action an agent should take, depending on an action-value role. This determines the significance of remaining in a particular state and completing a specific activity at that state depending on an action-value role.

It is one of the most meaningful progress in reinforcement learning by developing an algorithm to limit off-policy temporary deviation. Q-Learning measures a state-action value function for a target system that decides the highest value in picking the action. Function Q accepts the information as a current state (S) and action (A) and returns an estimated reward for that action in that state. Thus, q functions provide the arbitrary fixed values in the beginning before investigating the situation.

In this work, the following symptoms were considered to predict COVID-19 cases: fever, tiredness, dry cough, difficulty breathing, sore throat, pains, nasal congestion, runny nose, and diarrhea. Groups of symptoms are considered states, and action is taken based on the states. Here, the action is taken based on the increments in the confirmed and death cases. A reward is received when the foresight of verified cases and death cases are accurate. The action-value function defined as:


(10) $$\begin{array}{l} q\left(s,a;\theta \right)\approx Q^{*}\left(s,a\right) \end{array}$$

where q (s, a) represents the neural network approximation and θ is a reference variable representing the network's edge weights. The input to the neural network is a state, and the outputs for unconnected activities *Q={q(s,a*,θ*)|a*ϵ*A}s* are approximate *q* values.

The system is trained by depreciating forecast faults of *q*(*s, a*; θ). The DRL agent taking action at time t is *a*_*t*_ = argmax_*a*_*q*(*s*_*t*_, *a*; θ), where *q*(*s*_*t*_, *a*; θ) for various activities are supplied through the outcomes of the network. For example, assume that the resulting compensation is r_t+1_ and the state move to s_t+1_, then various actions are provided by the outputs of QNN. Consider that the resulting reward is r_t+1_ and the state moves to s_t+1_, then (s_t_,a_t_,r_t+1_,s_t+1_) establishes an “experience sample” that might be used to train the network. For training, the prediction error of network for the particular experience sample (s_t_,a_t_,r_t+1_,s_t+1_) is defined as:


(11) $$\begin{array}{l} L_{s_{t}},a_{t},r_{t+1},s_{t+1}\left(\theta \right)={\left(y_{r_{t+1}},s_{t+1}-q\left(s_{t},a_{t};\theta \right)\right)}^{2} \end{array}$$

Where θ = *weights*; and (*y*_*r*_*t*+1__, *s*_*t*+1_) = *targetoutput*, which is defined as:


(12) $$\begin{array}{l} \left(y_{r_{t+1}},s_{t+1}\right)=r_{t+1}+\gamma \underset{a^{\prime }}{\max}q\left(s_{t+1},a^{\prime };\theta \right) \end{array}$$

## Results and Discussions

This segment displays the trial setup and evaluation manner of the suggested prognostication design. Data of COVID-19 cases in India from Kaggle were employed with the Java framework to authenticate and examine the recommended standard.

The dataset utilized in the report comprises summary tables of regular time scales, including the number of reported cases and deaths over the past number of days from which the pandemic began. Data from January 30, 2020 (when the first case of COVID-19 was registered in India) to August 16, 2020, were analyzed, with 75% data employed for practice and 25% for predictive and validation purposes. Table 3 shows the sample data of confirmed and death cases daily and weekly.

**Table 3:** Daily and weekly sample data for COVID-19.

**Date**	**Confirmed**	**Deaths**	**Weekly confirmed**	**Weekly deaths**
30–01–20	1	0	1	0
31–01–20	0	0	1	0
01–02–20	0	0	1	0
02–02–20	1	0	2	0
…	…	…	…	…
14–08–20	64,553	1,007	434,116	6,455
15–08–20	65,002	996	437,581	6,518
16–08–20	63,490	944	436,672	6,601

**Figure 4:**
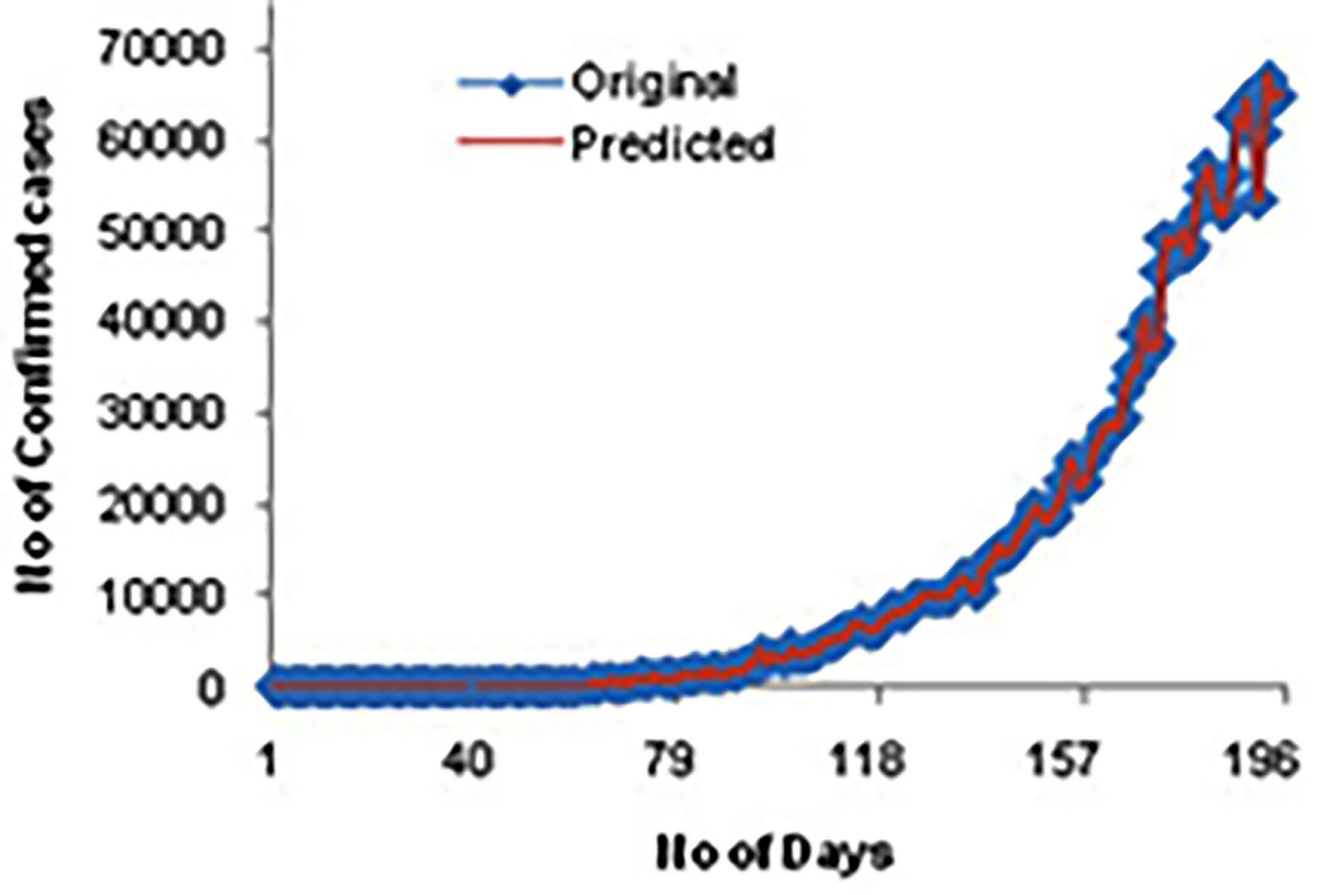
A confirmed case for original and predicted values.

Figure 4 shows the comparison of confirmed cases for the original and predicted values for 200 days. Here, we could see a high correlation between the actual and predicted cases.

**Figure 5:**
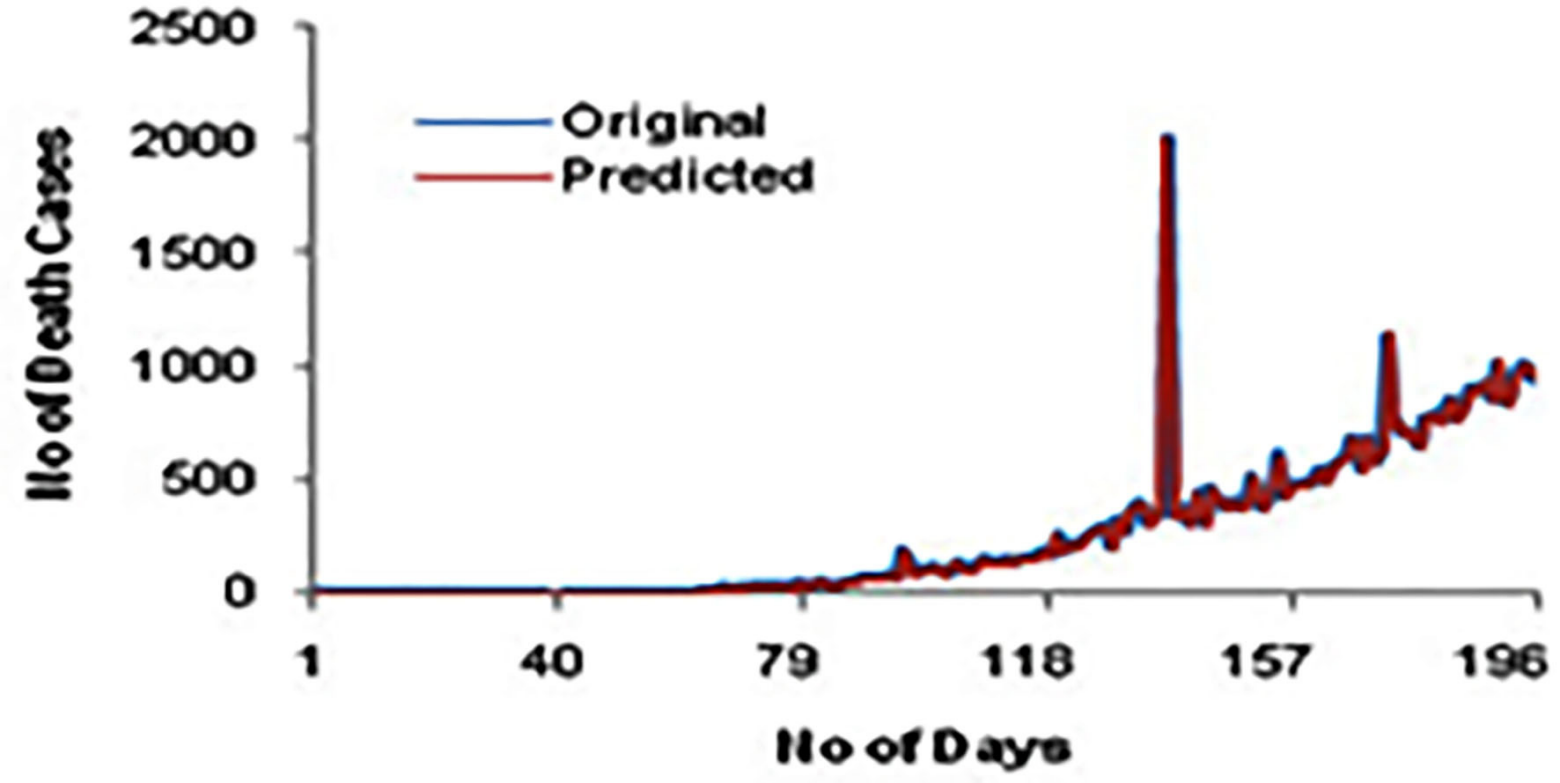
Predicted values for death cases.

Figure 5 shows the comparison of death cases for original and predicted values. It can be seen that the death rate slightly increased on specific days.

**Figure 6:**
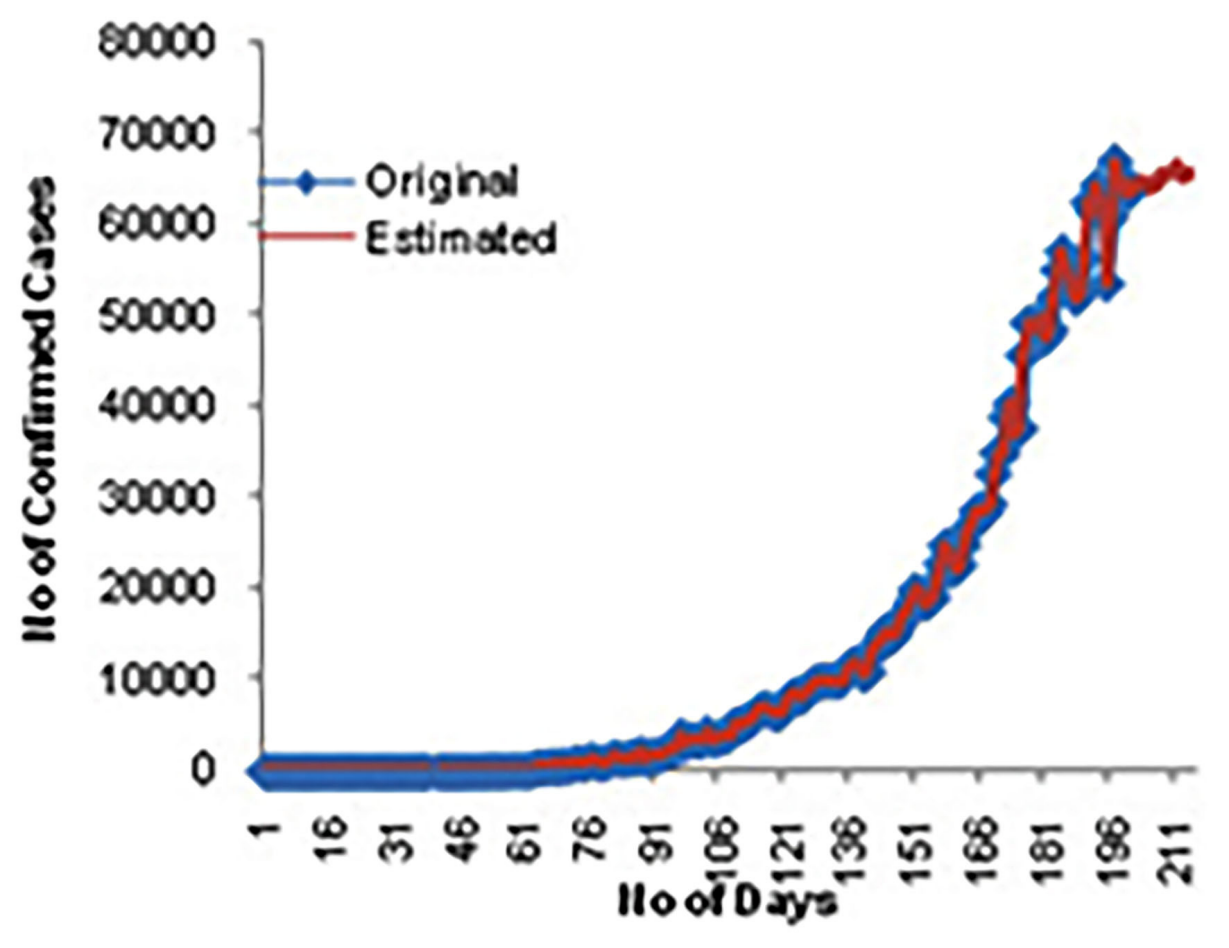
Estimated confirmed cases for 15 days.

**Figure 7:**
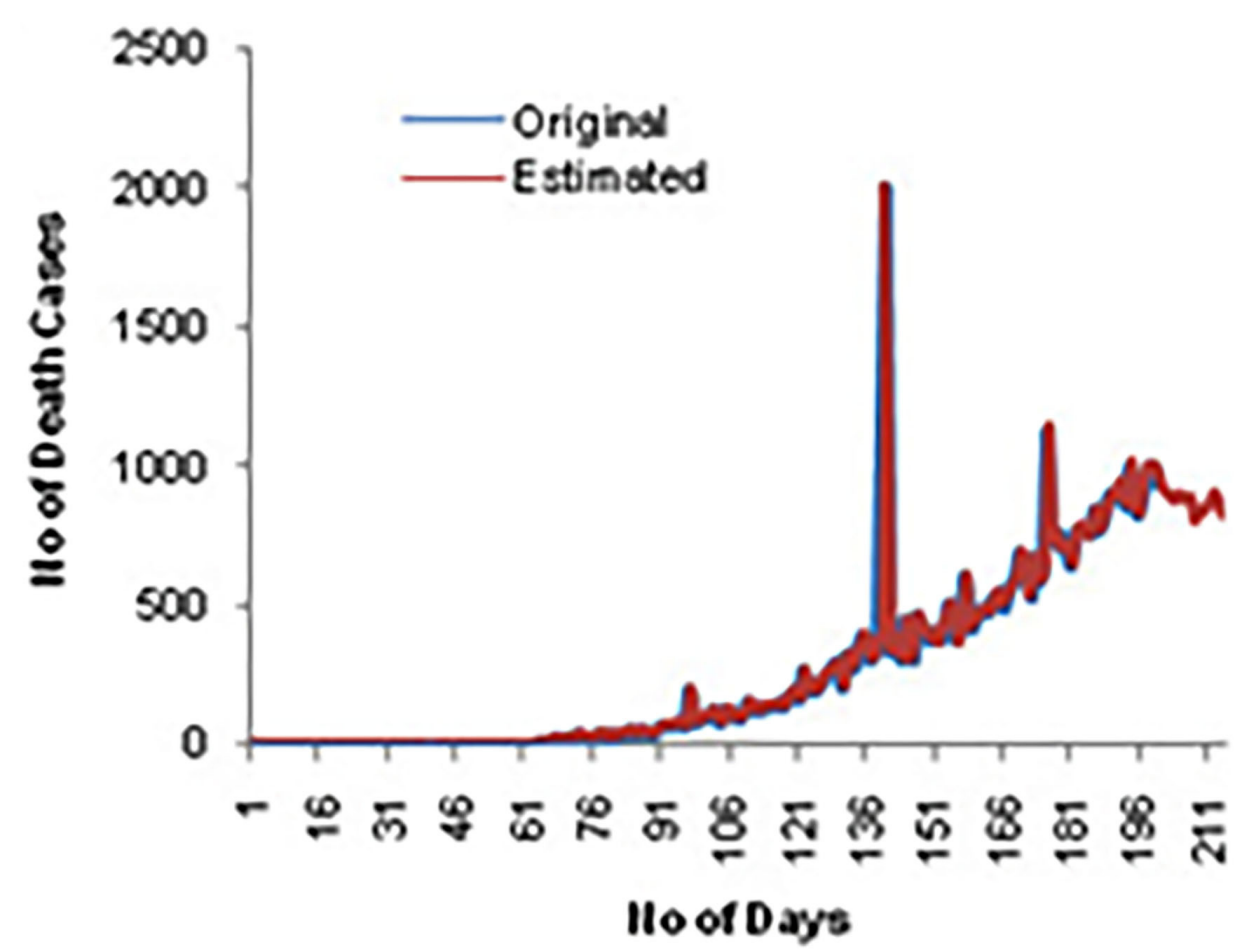
Death case estimated for next 15 days.

Figure 6 shows the estimated confirmed cases for the next 15 days. Figure 7 displays the estimated death cases for the next 15 days.

**Figure 8:**
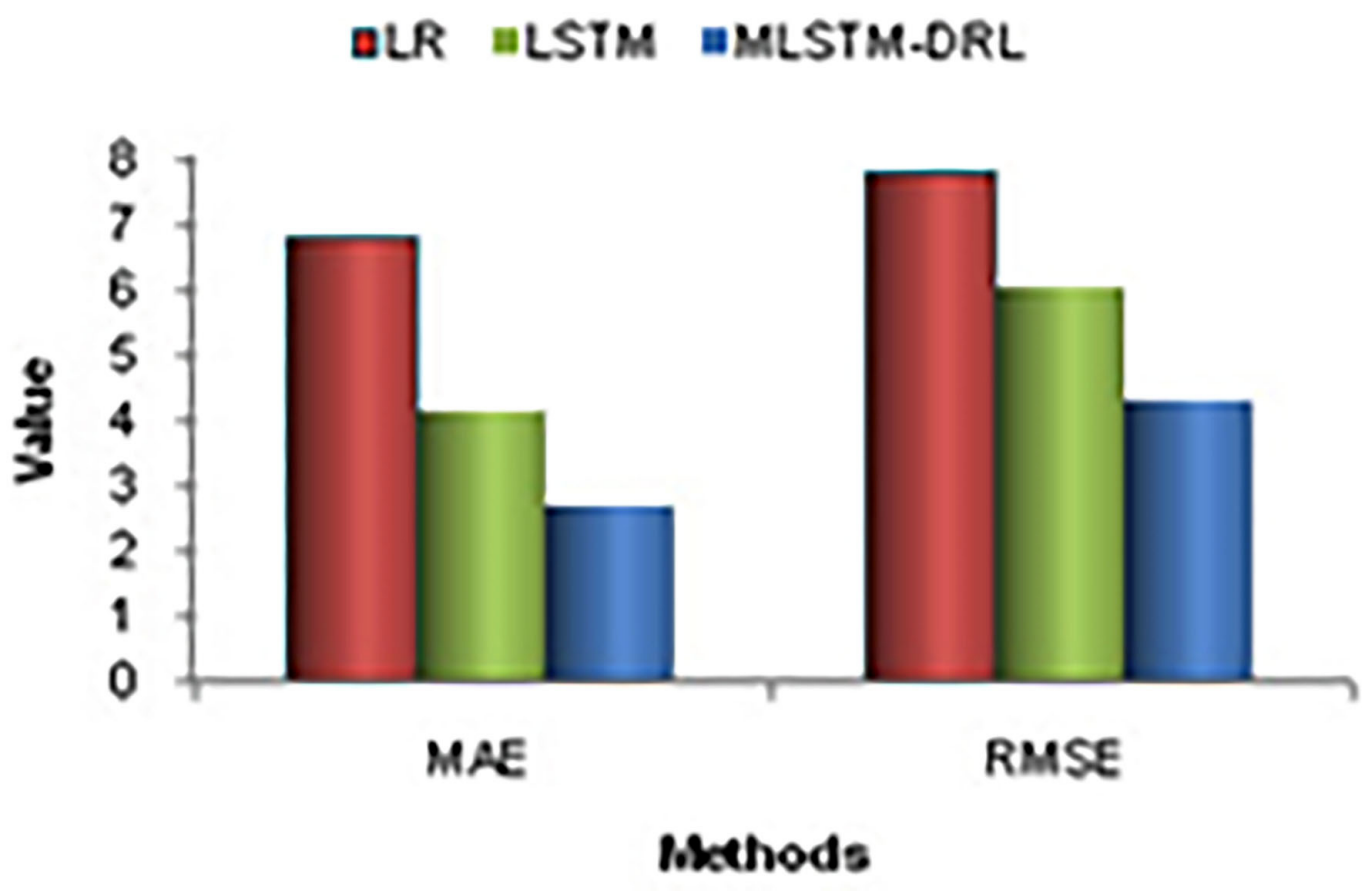
Comparison of evaluation metrics.

The proposed algorithm can evaluate the performance of learning in terms of the following metrics: Mean Absolute Error (MAE), Mean Square Error (MSE), and Root Mean Square Error (RMSE). Figure 8 shows the comparison of evaluation metrics. Based on the outcomes, the recommended MLSTM-DRL has a lowest error rate matched to other systems. We also see the ML model, Logistic Regression (LR) obtained the highest error rate. Deep learning methods are utilized to develop a system for future prediction of the COVID-19 affected cases. The study performs predictions on confirmed and death cases. It is a troubling circumstance for the world day by day as death and reported cases are rising. The number of individuals in various countries affected by the COVID-19 pandemic is not well-known. This analysis attempts to estimate the number of individuals who will be affected over the next 15 days in terms of freshly authenticated cases and deaths.

Individuals being affected by COVID-19 get increased daily, and the death rate has many ups and downs. The correlation between original and predicted data of confirmed and death cases are 0.999. The future prediction results can assist governments in planning lockdown or other medical decisions.

## Conclusion

COVID-19 is an ongoing pandemic that significantly endangers the health of people worldwide in a short period. A DL-based prediction method for forecasting the risk of COVID-19 has been proposed in this work. The framework analyses the actual day-to-day data dataset and uses deep learning algorithms to make predictions for upcoming days. This study determines the best activation function for M-LSTM; specifically, a deep reinforcement learning algorithm to optimize the prediction results. The proposed approach was compared with widely used existing algorithms like LR and LSTM. The finding of this work proves that the DL method can efficiently predict future cases of COVID-19. Overall, it can be concluded that the model's predictions are at per with the status of the virus; this may help understand and curb the spread of the virus.

Therefore, this study's forecast may be of great help in taking timely actions and making decisions to tackle the COVID-19 crisis. In the future, we advise using a semi-supervised hybrid design to identify COVID-19 and social media platforms to prevent further spread. It is also planned to publish the predicted results as a dashboard through Google Data Cloud.

## Data Availability Statement

The original contributions presented in the study are included in the article/supplementary material, further inquiries can be directed to the corresponding authors.

## Author Contributions

All authors listed have made a substantial, direct and intellectual contribution to the work, and approved it for publication.

## Funding

Open Access Funding by the Publication Fund of the TU Dresden.

## Conflict of Interest

The authors declare that the research was conducted in the absence of any commercial or financial relationships that could be construed as a potential conflict of interest.

## Publisher's Note

All claims expressed in this article are solely those of the authors and do not necessarily represent those of their affiliated organizations, or those of the publisher, the editors and the reviewers. Any product that may be evaluated in this article, or claim that may be made by its manufacturer, is not guaranteed or endorsed by the publisher.
